# Association between 5-year change in cardiovascular risk and the incidence of atherosclerotic cardiovascular diseases: a multi-cohort study

**DOI:** 10.1186/s12967-023-04488-7

**Published:** 2023-09-02

**Authors:** Jiayi Yi, Lili Wang, Xinli Guo, Xiangpeng Ren

**Affiliations:** 1 Department of Biochemistry, Medical College, Jiaxing University, No.899 Guangqiong Road, Jiaxing, 314001 Zhejiang China; 2 Department of Cardiology, Fuwai Hospital, Chinese Academy of Medical Sciences and Peking Union Medical College, National Center for Cardiovascular Diseases, Beijing, China

**Keywords:** Cardiovascular risk, Atherosclerotic cardiovascular disease, Primary prevention, Interventions

## Abstract

**Background:**

The influence of the historical cardiovascular risk status on future risk of atherosclerotic cardiovascular disease (ASCVD) is poorly understood. We aimed to investigate the association between 5-year changes in cardiovascular risk and ASCVD incidence.

**Methods:**

We analyzed pooled data from seven community-based prospective cohort studies with up to 20 years of follow-up data. The study populations included White or Black participants aged 40–75 years without prevalent ASCVD. Cardiovascular risk was assessed using the pooled cohort equation and was categorized into non-high (< 20%) or high risk (≥ 20%). Changes in cardiovascular disease (CVD) risk over a 5-year interval were recorded. The main outcome was incident ASCVD.

**Results:**

Among 11,026 participants (mean [SD] age, 60.0 [8.1] years), 4272 (38.7%) were female and 3127 (28.4%) were Black. During a median follow-up period of 9.9 years, 2560 (23.2%) ASCVD events occurred. In comparison with individuals showing a consistently high CVD risk, participants whose CVD risk changed from non-high to high (hazard ratio [HR], 0.67; 95% confidence interval [CI] 0.59–0.77) or high to non-high (HR, 0.57; 95% CI 0.41–0.80) and those with a consistently non-high risk (HR, 0.33; 95% CI 0.29–0.37) had a lower risk of incident ASCVD. In comparison with individuals showing a consistently non-high CVD risk, participants whose CVD risk changed from high to non-high (HR, 1.74; 95% CI 1.26–2.41) or from non-high to high risk (HR, 2.04; 95% CI 1.84–2.27) and those with a consistently high risk (HR 3.03; 95% CI 2.69–3.42) also showed an increased risk of incident ASCVD.

**Conclusions:**

Individuals with the same current CVD risk status but different historical CVD risks exhibited varying risks of future ASCVD incidents. Dynamic risk evaluation may enable more accurate cardiovascular risk stratification, and decision-making regarding preventive interventions should take the historical risk status into account.

**Graphical Abstract:**

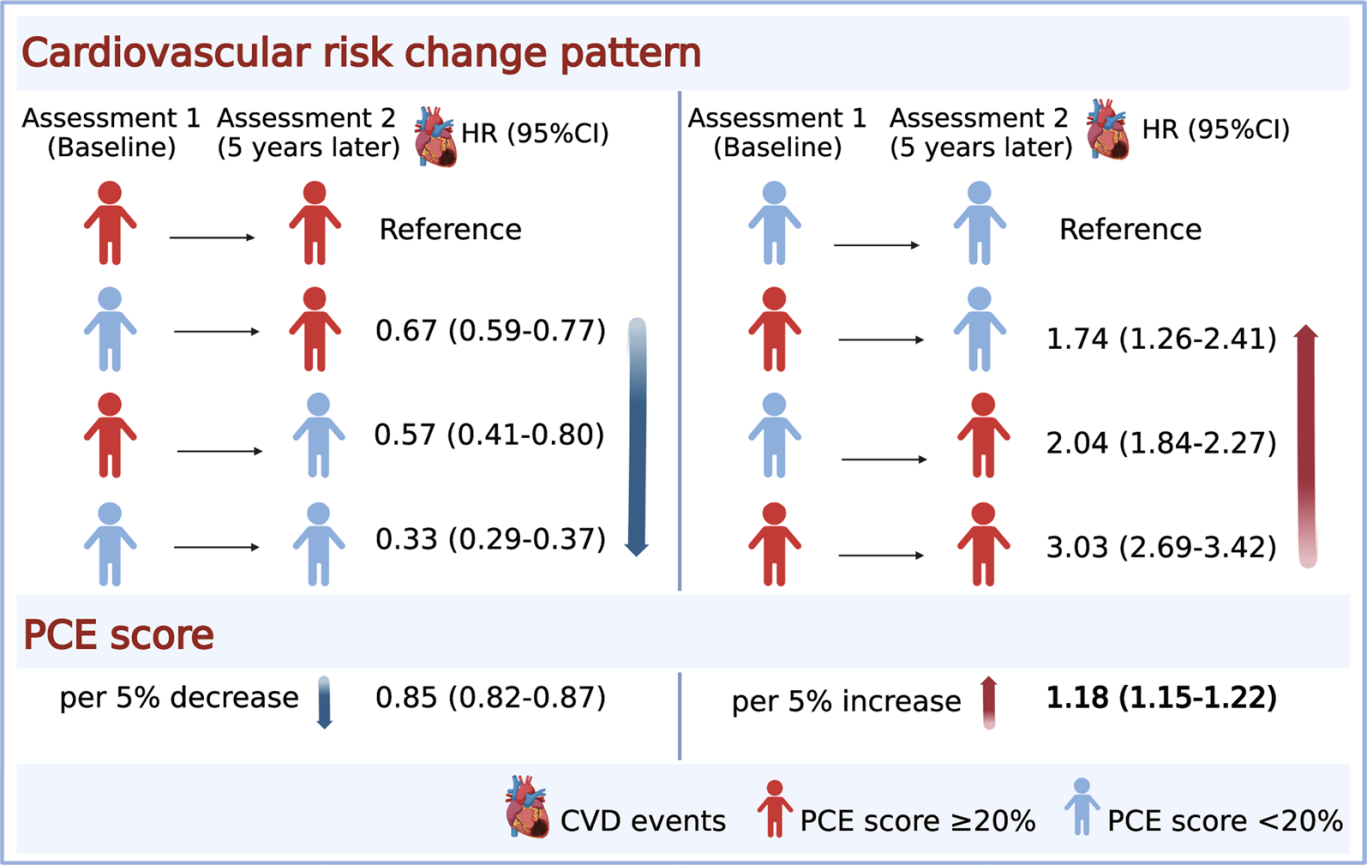

**Supplementary Information:**

The online version contains supplementary material available at 10.1186/s12967-023-04488-7.

## Introduction

Despite decades of declining incidence rates and significant improvements in management and outcomes, atherosclerotic cardiovascular disease (ASCVD) remains the major culprit for global morbidity and mortality [1–3]. The immense health and economic burdens caused by ASCVD can be attributed to the unsatisfactory implementation of prevention strategies and uncontrolled modifiable risk factors [4, 5]. Current preventive guidelines suggest routine assessments of ASCVD risk every 5 years in middle-aged and older-aged populations and formulation of appropriate interventions based on the latest risk factor measurement [6, 7]. Although risk-reducing strategies are essential for patients with a high risk of ASCVD, for individuals with borderline or intermediate risk, the value of preventive therapy remains uncertain, and patients may be reluctant to adhere to pharmacological therapy without clearer evidence of increased risk given the current risk evaluation.

Early measurements of ASCVD risk factors can provide valuable information about an individual’s risk history. The results of longitudinal measurements of blood lipid profiles and blood pressure in early adulthood have been shown to be associated with middle-aged cardiovascular events [8, 9]. Furthermore, several studies have shown that the patterns or trajectories of ASCVD risk factors change over time and are associated with varying levels of cardiovascular disease (CVD) risk [10–13]. Since lifestyle and medical interventions alter ASCVD risk, the use of historical risk assessment data could potentially improve risk stratification beyond that achieved with a single updated assessment while also enabling the setting of targets for preventive interventions.

Nevertheless, no previous study has clarified whether patients with the same current risk status but different historical risk factors have varied risks of future ASCVD. Improving our understanding of this issue could facilitate establishment of personalized goals for risk factor levels during future health evaluations while taking into account the influence of advancing age. In this study, we constructed a pooled cohort from seven large community-based longitudinal cohort studies in the U.S. to assess the association between a 5-year change in ASCVD risk and subsequent ASCVD incidence.

## Methods

### Study design and population

**Fig. 1:**
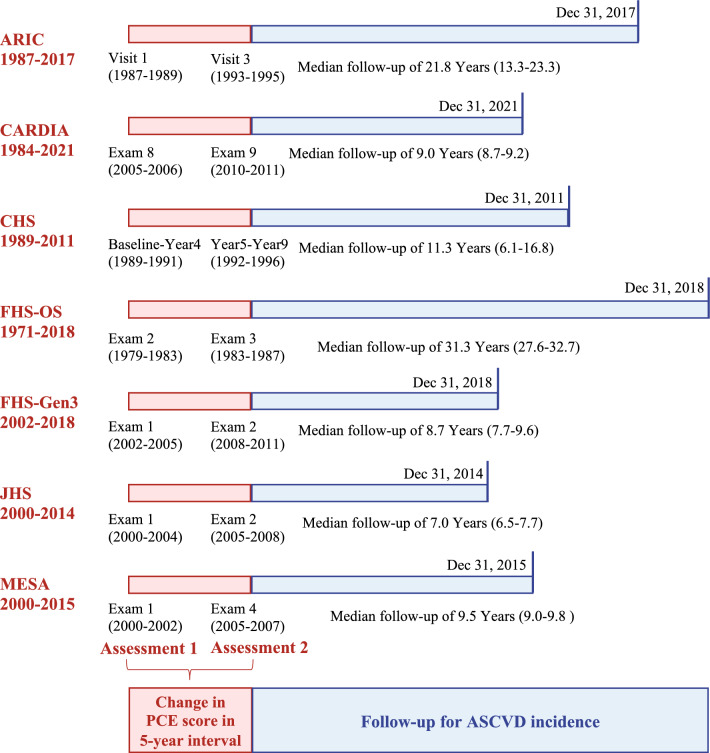
Study design. Schematic diagram showing analyses of baseline risk scores and changes in risk scores, by cohort, and follow-up for ASCVD events. *ARIC* Atherosclerosis Risk in Communities, *CARDIA* Coronary Artery Risk Development in Young Adults, *CHS* Cardiovascular Health Study, *FHS-OS* Framingham Heart Study Offspring Cohort, *FHS-Gen3* Framingham Heart Study Generation 3 Cohort, *JHS* Jackson Heart Study, *MESA* Multi-Ethnic Study of Atherosclerosis, *ASCVD* atherosclerotic cardiovascular disease

This study used individual-level data from seven community-based longitudinal cohort studies in the U.S., including Atherosclerosis Risk in Communities (ARIC) [14], Coronary Artery Risk Development in Young Adults (CARDIA) [15], Cardiovascular Health Study (CHS) [16], Framingham Heart Study (FHS) Offspring cohort [17], FHS Generation 3 cohort [17], Jackson Heart Study (JHS) [18], and Multi-Ethnic Study of Atherosclerosis (MESA) [19]. Participant consent and research ethics approvals were obtained for each cohort. The study designs and participant cohort characteristics, including the criteria for these cohorts, have been described elsewhere [14–19]. Notably, approximately one-third of the individuals in JHS also participated in the ARIC study and were only included once as part of the ARIC cohort [18]. For analyses of changes in ASCVD risk scores, baseline data and the corresponding data from examinations conducted 5 years later were used along with the latest available follow-up information in each cohort. The design of the current study is summarized in Fig. 1. This study was conducted in accordance with the Strengthening the Reporting of Observational Studies in Epidemiology (STROBE) reporting guideline [20].

On the basis of the derived study of pooled cohort equation (PCE) and guideline recommendations, among the 44,548 participants from the seven cohorts, we identified 31,574 White or Black individuals aged 40–75 years without prevalent ASCVD, which was defined as self-reported or examination-assessed coronary artery disease (angina or myocardial infarction) or ischemic or hemorrhagic stroke. The exclusion criteria were as follows: (1) missing variables for the 2-assessment calculation of PCE scores (n = 7149); (2) lost to follow-up (n = 21); (3) low ASCVD risk at baseline (PCE score < 5%; n = 12,880); and (4) ASCVD incidence within the 5-year period (n = 498). The remaining 11,026 individuals with borderline or higher baseline ASCVD risk (≥ 5%) were included for the analysis in this study (Additional file 1: Figure S1).

### Data collection

Self-reported questionnaires provided data on age, sex, race (Black, White), educational attainment (high school or below, college or above), family income (< $50,000/year, ≥ $50,000/year), current smoking status, antihypertensive medication or statin use. Weight, height, and systolic blood pressure were measured by physical examinations, and body mass index (BMI) was calculated as weight (kg) divided by the square of height (m^2^). Diabetes status was defined as either self-reported diabetes history, medication use, or fasting glucose ≥ 140 mg/dL [21]. Biochemistry profiles, including total cholesterol (TC), high-density lipoprotein cholesterol (HDL-C), high-sensitivity C-reactive protein (hs-CRP), and creatinine levels, were measured from fasting serum samples using similar approaches [14–19]. The estimated glomerular filtration rate (eGFR) was calculated using the Chronic Kidney Disease Epidemiology Collaboration (CKD-EPI) Eq.  [22].

### Assessments of ASCVD risk and change patterns

The ASCVD risk was assessed using the unmodified PCE (without refitting the model coefficients to the current dataset) and was derived using age, sex, race, TC and HDL-C levels, systolic blood pressure (SBP), antihypertensive therapy status, current smoking status, and diabetes status [23]. PCE scores ranged from 0 to 100%, and higher scores indicated higher risk. The threshold for high (≥ 20%) or non-high (< 20%) CVD risk was determined by current guideline recommendations [5]. The PCE score was measured at two selected 5-year interval assessments in each cohort (Fig. 1). Accordingly, we defined four CVD risk score change patterns: (1) consistently high risk (≥ 20% at both assessments); (2) change from non-high to high risk (< 20% to ≥ 20%); (3) change from high to non-high risk (≥ 20% to < 20%); (4) consistently non-high risk (< 20% at both assessments).

### Outcomes

The primary outcome of this study was incident ASCVD, defined as incident nonfatal myocardial infarction, fatal coronary heart disease, or fatal or nonfatal stroke during follow-up. The definitions, attainment, and adjudication procedures of study outcomes were formulated and implemented by an expert panel for each cohort, and detailed descriptions of this information have been published previously [14–19]. For our pooled analysis, we included all ASCVD events from the second assessment through the latest available follow-up for each individual study cohort.

### Statistical analysis

The baseline characteristics were reported as mean (standard deviation, SD), median (interquartile range, IQR), or number (percentage) and were compared using t-tests, standard nonparametric tests, or χ^2^ tests as appropriate. Cox proportional-hazard models were used to determine hazard ratios (HRs) and the corresponding 95% confidence intervals (CIs) for the associations of the risk change patterns and PCE score change values with the ASCVD incidence. Schoenfeld residuals were used to test the proportional-hazards assumption, and no obvious violation was observed [24]. We constructed three Cox models in this study: (1) an unadjusted model, (2) a model adjusted for educational attainment and family income, and (3) a model additionally adjusted for BMI, statin use, hs-CRP level, and eGFR. To examine subpopulations susceptible to demographic-related disparities, stratified analyses were performed by age (40–59 and 60–75 years), sex, and race. The P values for the production terms between risk score change patterns and the stratified factors were used to estimate the significance of interactions. To assess the robustness of our findings, we also performed sensitivity analyses by (1) excluding ASCVD events that occurred during the first 3-year follow-up period to reduce the possibility of reverse causation and (2) excluding participants with borderline (5–7.5%) ASCVD risk at baseline. Statistical tests were 2-sided, and statistical significance was set at P < 0.05. All statistical analyses were conducted between December 2022 and March 2023 using R software, version 4.2.0 (R Core Team, Vienna, Austria).

## Results

### Baseline characteristics

A total of 11,026 participants were included in the final analysis. The mean (SD) age of the study population at baseline was 60.0 (8.1) years. Among the 11,026 participants, 4272 (38.7%) were female and 3127 (28.4%) were Black (Table 1). The mean (SD) interval between the two assessments was 5.4 (0.7) years. The median (IQR) baseline PCE score of the study population was 10.4% (7.1%-15.8%). A total of 1760 (16.0%) individuals were identified as showing high ASCVD risk at baseline, and 221 (2.0%) of them showed non-high risk at the second assessment. On the other hand, 7036 (63.8%) individuals consistently showed non-high risk during the 5-year period, while 2230 (20.2%) individuals showed progression to high risk from non-high risk at baseline. The baseline characteristics of the overall study cohort and participants categorized by ASCVD risk change are summarized in Table 1, while categorization by cohort is presented in Additional file 1: Table S1. In general, participant characteristics varied across ASCVD risk change categories (all P < 0.01). In comparison with individuals showing consistently high risk, those in the other three categories were younger and more likely to be women, have a better educational background, and have higher family income (Table 1).

**Table 1:** Baseline characteristics of the study population by the 5-year ASCVD risk change patterns

Characteristics	Total (N = 11,026)	Consistently high (n = 1539)	Non-high to high (n = 2230)	High to non-high (n = 221)	Consistently non-high (n = 7036)
Age, mean (SD), years	60.0 (8.1)	67.2 (6.3)	63.2 (7.3)	62.6 (7.3)	57.3 (7.3)
Female, n (%)	4272 (38.7)	424 (27.6)	958 (43.0)	83 (37.6)	2807 (39.9)
Black race, n (%)	3127 (28.4)	425 (27.6)	546 (24.5)	111 (50.2)	2045 (29.1)
Education attainment, n (%)^a^					
High school or below	5247 (47.6)	794 (51.6)	1132 (50.8)	90 (40.7)	3231 (45.9)
College or above	5672 (51.4)	738 (48.0)	1092 (49.0)	127 (57.5)	3715 (52.8)
Family income, n (%)^a^					
< 50,000 $/year	7288 (66.1)	1141 (74.1)	1625 (72.9)	139 (62.9)	4383 (62.3)
≥ 50,000 $/year	3083 (28.0)	292 (19.0)	486 (21.8)	66 (29.9)	2239 (31.8)
BMI, mean (SD), kg/m^2^	28.5 (5.1)	28.9 (4.9)	28.5 (5.1)	29.6 (5.5)	28.5 (5.2)
ASCVD risk factors					
SBP, mean (SD), mmHg	129.7 (18.6)	144.2 (20.4)	130.3 (16.7)	144.5 (21.3)	125.9 (16.8)
Antihypertensive medication, n (%)	4202 (38.1)	934 (60.7)	911 (40.9)	150 (67.9)	2207 (31.4)
Diabetes, n (%)	1522 (13.8)	567 (36.8)	309 (13.9)	75 (33.9)	571 (8.1)
Current smoker, n (%)	3010 (27.3)	411 (26.7)	459 (20.6)	99 (44.8)	2041 (29.0)
Total cholesterol, mean (SD), mg/dL	212.7 (41.6)	207.5 (43.2)	212.0 (40.1)	215.5 (47.8)	214.0 (41.4)
HDL cholesterol, mean (SD), md/dL	48.1 (14.8)	45.9 (14.1)	49.2 (15.5)	46.7 (13.9)	48.2 (14.6)
Baseline PCE score, median [IQR], %	10.4 [7.1, 15.8]	25.3 [22.1, 31.3]	13.8 [11.1, 16.5]	22.4 [20.7, 25.7]	8.0 [6.3, 10.7]
Statin usage, n (%)	864 (7.8)	181 (11.8)	161 (7.2)	21 (9.5)	501 (7.1)
Hs-CRP, median [IQR], mg/L	2.1 [1.0, 4.5]	2.4 [1.1, 4.5]	2.3 [1.1, 4.7]	2.3 [1.0, 4.6]	1.9 [0.9, 4.3]
eGFR, mean (SD), mL/min/1.73 m^2^	90.2 (19.6)	81.1 (19.6)	88.7 (19.0)	85.8 (22.8)	92.7 (19.1)
Assessments time interval, mean (SD), years	5.4 (0.7)	5.1 (0.6)	5.4 (0.7)	5.1 (0.6)	5.4 (0.7)

*ASCVD* atherosclerotic cardiovascular disease, *SD* standard deviation, *BMI* body mass index, *SBP* systolic blood pressure, *HDL-C* high-density lipoprotein cholesterol, *PCE* pooled cohort equation, *IQR* interquartile range, *hs-CRP* high-sensitivity C-reactive protein, *eGFR* estimated glomerular filtration rate

^a^The percentage may not sum to 100% because of missing data

**Fig. 2:**
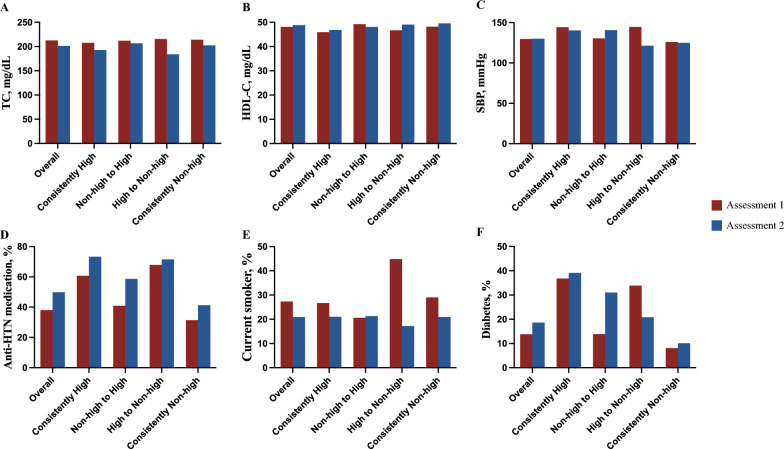
Change in cardiovascular risk factors including **A** TC, **B** HDL-C, **C**SBP, **D** anti-HTN medication, **E** smoking, **F** diabetes over 5 years. *TC* total cholesterol, *HDL-C* high-density lipoprotein cholesterol, *SBP* systolic blood pressure, *HTN* hypertension

The distribution of modifiable cardiovascular risk factors in the two assessments has been summarized in Fig. 2. Among participants whose CVD risk changed from high to non-high, the proportions of current smokers (from 44.8 to 17.2%) and patients with diabetes (from 33.9 to 20.8%) substantially reduced during the two assessments periods. These participants also showed reductions in the TC level (from 215.5 to 184.1 mg/dL) and SBP (from 144.5 to 121.3 mmHg), while their HDL-C levels increased slightly (from 46.7 to 49.0 mg/dL). Among participants whose CVD risk changed from non-high to high, the proportion of patients with diabetes increased substantially (from 13.9 to 31.0%). Despite the increased usage of antihypertensive medication (from 40.9 to 58.6 mmHg), the SBP in this group increased (from 130.3 to 140.7 mmHg) during the 5-year interval.

### 5-Year CVD risk change and incident ASCVD

During the median follow-up period of 9.9 years, 2560 (23.2%) ASCVD incidents were observed. The incidence of ASCVD corresponding to each risk change category has been described in Table 2. In multivariable analysis, the risk of ASCVD was lower in individuals whose CVD risk changed from non-high to high (HR, 0.67; 95% CI 0.59–0.77), from high to non-high (HR, 0.57; 95% CI 0.41–0.80), and remained consistently non-high (HR, 0.33; 95% CI 0.29–0.37), in comparison with individuals who showed consistently high risk (Table 2). In comparison with individuals showing consistently non-high risk, participants whose CVD risk changed from high to non-high (HR, 1.74; 95% CI 1.26–2.41), from non-high to high (HR, 2.04; 95% CI 1.84–2.27), and remained consistently high (HR, 3.03; 95% CI 2.69–3.42) all showed an elevated risk of future ASCVD. In the stratified analysis, younger, female, and Black individuals whose CVD risk changed from high to non-high did not show a significant association with reduced risk of ASCVD (P > 0.05) in comparison with the consistently high group. Similarly, in comparison with the individuals showing a consistently non-high risk, older and female participants whose CVD risk changed from high to non-high did not show a significantly increased risk of ASCVD (P > 0.05; Additional file 1: Figures S2 and S3).

**Table 2:** Associations between change patterns in the 5-year ASCVD risk status and subsequent ASCVD incidence

	Events/Total	Unadjusted model		Multivariable model 1^a^		Multivariable model 2^b^	
		HR (95% CI)	*p* value	HR (95% CI)	*p* value	HR (95% CI)	*p* value
ASCVD risk change group (with consistently high as the reference)							
Consistently High	519/1539	1 (Reference)	/	1 (Reference)	/	1 (Reference)	/
Non-high to High	681/2230	0.66 (0.59–0.74)	< 0.01	0.66 (0.59–0.75)	< 0.01	0.67 (0.59–0.77)	< 0.01
High to non-high	51/221	0.62 (0.46–0.82)	< 0.01	0.63 (0.46–0.85)	< 0.01	0.57 (0.41–0.80)	< 0.01
Consistently non-high	1309/7036	0.31 (0.28–0.35)	< 0.01	0.32 (0.29–0.36)	< 0.01	0.33 (0.29–0.37)	< 0.01
ASCVD risk change group (with consistently non-high as the reference)							
Consistently non-high	1309/7036	1 (Reference)	/	1 (Reference)	/	1 (Reference)	/
High to non-high	51/221	1.97 (1.49–2.61)	< 0.01	1.94 (1.45–2.61)	< 0.01	1.74 (1.26–2.41)	< 0.01
Non-high to high	681/2230	2.11 (1.93–2.33)	< 0.01	2.05 (1.86–2.26)	< 0.01	2.04 (1.84–2.27)	< 0.01
Consistently high	519/1539	3.19 (2.88–3.54)	< 0.01	3.09 (2.77–3.44)	< 0.01	3.03 (2.69–3.42)	< 0.01

*ASCVD* atherosclerotic cardiovascular disease, *HR* hazard ratio, *CI* confidence interval

^a^Adjusted for educational attainment and family income

^b^Additionally adjusted for body mass index, high-sensitivity C-reactive protein, statin usage, and estimated glomerular filtration rate

**Fig. 3:**
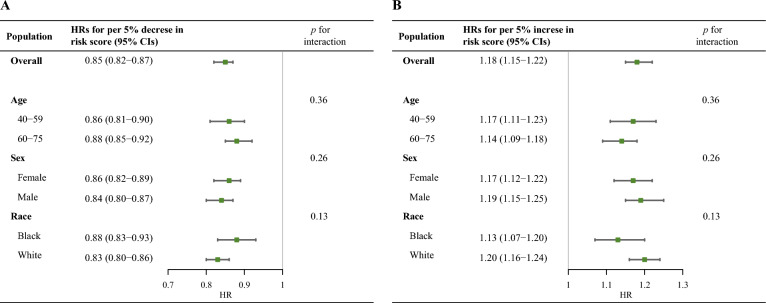
Hazard ratios and 95% confidence intervals for incident atherosclerotic cardiovascular disease per 5% decrease (**A**) or increase (**B**) in the PCE risk score over 5-year intervals stratified by age, sex, and race. All HRs were adjusted for educational attainment, family income, body mass index, high-sensitivity C-reactive protein level, statin usage, and estimated glomerular filtration rate. *PCE* pooled cohort equation, *ASCVD* atherosclerotic cardiovascular disease, *HR* hazard ratio < *CI* confidence interval

Both reductions (HR for per 5% decrease, 0.85; 95% CI 0.82–0.87) and increments (HR for per 5% increase, 1.18; 95% CI 1.15–1.22) in the continuous PCE score over 5 years were associated with ASCVD risk, and the association was consistent among demographic subgroups (Fig. 3). However, reductions in the PCE score were not associated with decreased ASCVD risk in patients with a consistently high risk and those showing a change from high to non-high risk, and increased PCE scores were not associated with an increase in ASCVD risk in the patients showing a change from high to non-high risk and those with a consistently high risk (Additional file 1: Figures S4 and S5).

### Sensitivity analysis

The results were generally robust in sensitivity analyses. After excluding participants who showed ASCVD incidence within 3 years of follow-up or had borderline ASCVD risk at baseline, in comparison with the consistently high risk category, the reduction in ASCVD risk within the other three groups did not change significantly and remained evident. Similar results were also observed in the comparison with the consistently non-high risk category (Additional file 1: Tables S2 and S3).

## Discussion

In this analysis of longitudinal data from seven community-based large-scale cohorts, the ASCVD risk change patterns in a 5-year interval were associated with varying levels of ASCVD risk. Despite showing the same risk status in the latest assessment, individuals who transitioned from borderline or intermediate (5–20%) risk to high risk (≥ 20%) had lower odds of future ASCVD incidence than those with consistently high risk. Conversely, participants who transitioned from high to non-high CVD risk had a higher risk of ASCVD than those who consistently showed non-high risk. Both reductions and increments in the PCE score were associated with ASCVD incidents, and the association was generally consistent across demographic subgroups. Our results suggest that monitoring changes in PCE scores over time may enable more accurate cardiovascular risk stratification and that decision-making regarding preventive interventions should take historical risk status into consideration.

To the best of our knowledge, this is the first attempt to explore the relationship between CVD risk status changes and long-term ASCVD incidence. Lindbohm et al. have shown that adding the value of a 5-year change in the European Society of Cardiology’s Systematic Coronary Risk Evaluation (SCORE) or PCE score as a new predictor could improve the predictive performance of the original model. In addition, both increments and reductions in risk scores have been shown to be associated with CVD-free life-years [25]. The findings from this study implicated the value of longitudinal risk assessments in the primary prevention of CVD. However, this study did not examine the differences in ASCVD risk among different risk status change patterns.

The differences in ASCVD incidence risks among individuals with the same current risk status and different historical risk statuses can be attributed to the accumulated atherosclerotic burden. Subclinical atherosclerosis can develop much before the onset of evident cardiovascular disease, and the longer the atherosclerotic processes remain uncontrolled, the fewer opportunities there are for effective preventive interventions. This explanation is supported by evidence showing that the preventive effects of interventions such as smoking cessation and the use of antihypertensive or statin medications tend to diminish in the older population [26–28]. Previous studies have also shown cumulative effects of several CVD risk factors such as blood pressure, blood lipids, and smoking in the progression of atherosclerosis [29–31]. Thus, it is reasonable to assume that individuals transitioning from a lower risk status to high risk have a reduced risk of ASCVD in comparison with those showing a consistently high risk. This finding highlights the importance of lifetime efforts to maintain an optimal lifestyle and avoid progression into a high-risk state as early as possible. It also indicates that a stricter target for risk factor management might be required for individuals with longer high-risk exposure.

Improvements in the risk score would require a substantial effort in controlling modifiable CVD risk factors such as smoking cessation or blood pressure and cholesterol management since the changes in the risk score reflect the net effect of lifestyle modifications caused by risk factor changes in addition to increasing age between risk assessments. However, we found that the clinical benefit of these efforts varied among demographic subpopulations. Changing from high risk to borderline or intermediate risk in younger, female, and Black individuals was not associated with reduced ASCVD incidence risk in comparison with those having a consistently high risk, and in comparison with those having a consistently lower risk status, individuals changing from high risk to non-high risk still showed an increased ASCVD incidence risk in the younger or male populations. These results indicate that commonly recommended lifestyle interventions may be insufficient to effectively prevent or delay the onset of cardiovascular events in these populations even when they show a lower current estimated ASCVD risk.

In clinical scenarios, the value of preventive therapy remains uncertain for many individuals with non-high CVD risk, and some patients may be reluctant to take medical therapy without clearer evidence of an increased ASCVD risk [5]. Our results provide a new perspective on this issue. Patients with historically high CVD risk had an increased risk of future ASCVD incidence even if the latest single CVD risk assessment showed borderline or intermediate CVD risk. Therefore, more aggressive preventive interventions may be initialized in these individuals.

The results of the current study may have major implications for healthcare providers and public health professionals in their efforts to perform CVD prevention and management. First, by monitoring individuals’ risk status over time rather than referring to a single assessment, healthcare providers can develop more effective, precise, and personalized strategies for preventing and treating CVD. This finding highlights the importance of regular and comprehensive risk reassessments to ensure that preventive measures are developed and implemented in a timely manner. Second, for individuals with a high ASCVD risk, an improved risk status may not be predictive of a reduced risk of future CVD. This finding highlights the importance of lifetime efforts to avoid developing a high-risk profile.

This study had several strengths, including a relatively large, pooled cohort with a long follow-up period and repeated standardized risk factor measurements, which enabled us to examine changes in risk over time. In addition, we used the PCE score, the most widely used and recommended CVD risk assessment tool for the U.S. population, as the ASCVD risk assessment in the current study.

### Limitations

The following limitations also require consideration while interpreting our findings. First, several cohorts in this study (e.g., MESA, CARDIA, and ARIC) included participants healthier than the general population. Therefore, the prevalence and outcome incidence of risk factors may have been underestimated in comparison with those in the general population. Second, given the longitudinal nature of this study, differential loss to follow-up could have resulted in selection bias. Third, the CVD risks were assessed at two time points in a 5-year interval, and the categories of risk change may not have captured potential risk factor fluctuations between the assessments or during follow-up. Fourth, the results from this study may have been confounded by the lack of adjustment for some potential CVD-related clinical and laboratory parameters (e.g. diet and physical activity). Finally, although we controlled the results for several potential confounders, the nature of observational study designs precluded us from concluding causality between longitudinal risk change and ASCVD incidence.

## Conclusions

In this longitudinal multi-cohort study, within a 5-year period, transitioning from non-high CVD risk to high risk was related to reduced odds of future ASCVD incidents, while transitioning from high CVD risk to non-high risk was associated with an increased risk of ASCVD. Changes in PCE scores were consistently associated with ASCVD incidents across demographic subgroups. Future research examining the causality associations and mechanisms of longitudinal risk changes and incident ASCVD and validating and generalizing our findings in other populations is needed to further strengthen our understanding in this field.

### Supplementary Information

**Additional file 1: Figure S1. **Study Selection Flowchart. **Table S1.** Characteristics of Study Participants by Study Cohorts. **Figure S2. **Associations between the 5-year ASCVD risk status change patterns and subsequent ASCVD incidence stratified by age, sex, and race (with consistently high as the reference). **Figure S3.** Associations between the 5-year ASCVD risk status change patterns and subsequent ASCVD incidence stratified by age, sex, and race (with consistently non-high as the reference). **Figure S4.** Hazard ratios and 95% confidence intervals for incident atherosclerotic cardiovascular disease per 5% decrease in PCE risk score over 5-year intervals stratified by risk change patterns. **Figure S5.** Hazard ratios and 95% confidence intervals for incident atherosclerotic cardiovascular disease per 5% increase in PCE risk score over 5-year intervals stratified by risk change patterns. **Table S2. **Sensitivity analysis by excluding participants with ASCVD incidence that occurred during the first 3-year follow-up. **Table S3.** Sensitivity analysis by excluding participants with borderline risk (5-7.5%) at baseline

## Data Availability

Data and materials used in this study are available from the corresponding author upon reasonable request.

## Abbreviations

ARIC: Atherosclerosis risk in communities
ASCVD: Atherosclerotic cardiovascular disease
BMI: Body mass index
CARDIA: Coronary artery risk development in young adults
CHS: Cardiovascular health study
CI: Confidence interval
CVD: Cardiovascular disease
CVH: Cardiovascular health
FHS: Framingham heart study
HDL-C: High-density lipoprotein cholesterol
HR: Hazard ratio
IQR: Interquartile range
JHS: Jackson heart study
MESA: Multi-ethnic study of atherosclerosis
PCE: Pooled cohort equation
SBP: Systolic blood pressure
SCORE: Systematic coronary risk evaluation
TC: Total cholesterol
