# Supplementary material for: Association between 5-year change in cardiovascular risk and the incidence of atherosclerotic cardiovascular diseases: a multi-cohort study
Source: J Transl Med. 2023 Sep 2;21:589. doi: 10.1186/s12967-023-04488-7 (PMC10475181; doi:10.1186/s12967-023-04488-7)
Supplement: Supplementary file 1 — Additional file 1: Figure S1. Study Selection Flowchart. Table S1. Characteristics of Study Participants by Study Cohorts. Figure S2. Associations between the 5-year ASCVD risk status change patterns and subsequent ASCVD incidence stratified by age, sex, and race (with consistently high as the reference). Figure S3. Associations between the 5-year ASCVD risk status change patterns and subsequent ASCVD incidence stratified by age, sex, and race (with consistently non-high as the reference). Figure S4. Hazard ratios and 95% confidence intervals for incident atherosclerotic cardiovascular disease per 5% decrease in PCE risk score over 5-year intervals stratified by risk change patterns. Figure S5. Hazard ratios and 95% confidence intervals for incident atherosclerotic cardiovascular disease per 5% increase in PCE risk score over 5-year intervals stratified by risk change patterns. Table S2. Sensitivity analysis by excluding participants with ASCVD incidence that occurred during the first 3-year follow-up. Table S3. Sensitivity analysis by excluding participants with borderline risk (5-7.5%) at baseline [file 12967_2023_4488_MOESM1_ESM.docx]

**Additional file**

**Association between 5-year change in cardiovascular risk and atherosclerotic cardiovascular disease incidence: A multicohort study**

Jiayi Yi, MD^2^; Lili Wang, MD^2^; Xinli Guo^2^; MD, Xiangpeng Ren^1^, PhD

From ^1^ Department of Biochemistry, Medical College, Jiaxing University, Jiaxing, China. ^2^ Department of Cardiology, Fuwai Hospital, Chinese Academy of Medical Sciences and Peking Union Medical College, National Center for Cardiovascular Diseases, Beijing, China, and

**Contents**

Figure S1. Study Selection Flowchart

Table S1. Characteristics of Study Participants by Study Cohorts

Figure S2. Associations between the 5-year ASCVD risk status change patterns and subsequent ASCVD incidence stratified by age, sex, and race (with consistently high as the reference).

Figure S3. Associations between the 5-year ASCVD risk status change patterns and subsequent ASCVD incidence stratified by age, sex, and race (with consistently non-high as the reference).

Figure S4. Hazard ratios and 95% confidence intervals for incident atherosclerotic cardiovascular disease per 5% decrease in PCE risk score over 5-year intervals stratified by risk change patterns

Figure S5. Hazard ratios and 95% confidence intervals for incident atherosclerotic cardiovascular disease per 5% increase in PCE risk score over 5-year intervals stratified by risk change patterns

Table S2. Sensitivity analysis by excluding participants with ASCVD incidence that occurred during the first 3-year follow-up

Table S3. Sensitivity analysis by excluding participants with borderline risk (5-7.5%) at baseline

Figure S1. Study Selection Flowchart

Abbreviations: ARIC, Atherosclerosis Risk in Communities; CARDIA, Coronary Artery Risk Development in Young Adults; CHS, Cardiovascular Health Study; FHS-OS, Framingham Heart Study Offspring; FHS-Gen3 Framingham Heart Study Generation 3; JHS, Jackson Heart Study; MESA, Multi-Ethnic Study of Atherosclerosis; ASCVD, atherosclerotic cardiovascular disease; PCE, pooled cohort equation.

Table S1. Characteristics of Study Participants by Study Cohorts

| **Characteristics** | **ARIC**  **N=4999** | **CARDIA**  **N=378** | **CHS**  **N=1895** | **FHS-OS**  **N=379** | **FHS-GenIII**  **N=233** | **JHS**  **N=427** | **MESA**  **N=2715** |
| --- | --- | --- | --- | --- | --- | --- | --- |
| **Age, mean (SD), years** | 56.8 (5.3) | 46.7 (2.8) | 69.4 (2.5) | 52.4 (6.2) | 51.5 (6.3) | 55.8 (7.9) | 63.7 (7.4) |
| **Female, n (%)** | 1576 (31.5) | 80 (21.2) | 1159 (61.2) | 99 (26.1) | 29 (12.4) | 192 (45.0) | 1137 (41.9) |
| **Black Race, n (%)** | 1391 (27.8) | 273 (72.2) | 70 (3.7) | 0 (0.0) | 0 (0.0) | 427 (100.0) | 966 (35.6) |
| **Education attainment, n (%) ^a^** |  |  |  |  |  |  |  |
| High school or below | 2843 (56.9) | 194 (51.3) | 972 (51.3) | 144 (38.0) | 60 (25.8) | 45 (10.5) | 989 (36.4) |
| College or above | 2147 (42.9) | 160 (42.3) | 920 (48.5) | 171 (45.1) | 173 (74.2) | 382 (89.5) | 1719 (63.3) |
| **Family Income, n (%) ^a^** |  |  |  |  |  |  |  |
| < 50,000 $/year | 3698 (74.0) | 160 (42.3) | 1453 (76.7) | 214 (56.5) | 53 (22.7) | 172 (40.3) | 1538 (56.6) |
| ≥ 50,000 $/year | 1033 (20.7) | 210 (55.6) | 327 (17.3) | 91 (24.0) | 172 (73.8) | 183 (42.9) | 1067 (39.3) |
| **BMI, mean (SD), kg/m2** | 28.4 (5.0) | 31.0 (6.3) | 26.6 (3.9) | 27.3 (3.8) | 30.2 (5.8) | 32.4 (6.5) | 29.3 (5.1) |
| **ASCVD risk factors** |  |  |  |  |  |  |  |
| SBP, mean (SD) mmHg | 127.4 (18.0) | 132.3 (17.4) | 132.3 (19.3) | 130.9 (15.9) | 130.9 (14.8) | 131.0 (15.5) | 131.4 (19.9) |
| Antihypertensive medication**,** n (%) | 1673 (33.5) | 157 (41.5) | 696 (36.7) | 76 (20.1) | 66 (28.3) | 281 (65.8) | 1253 (46.2) |
| Diabetes**,** n (%) | 718 (14.4) | 71 (18.8) | 135 (7.1) | 18 (4.7) | 47 (20.2) | 114 (26.7) | 419 (15.4) |
| Current smoker**,** n (%) | 1671 (33.4) | 203 (53.7) | 243 (12.8) | 194 (51.2) | 96 (41.2) | 64 (15.0) | 539 (19.9) |
| Total cholesterol, mean (SD), mg/dL | 222.3 (42.0) | 193.1 (42.5) | 214.9 (37.7) | 231.2 (38.2) | 209.6 (38.9) | 205.9 (39.7) | 195.0 (37.0) |
| HDL cholesterol, mean (SD), md/dL | 45.7 (14.1) | 45.0 (13.9) | 55.4 (15.8) | 41.8 (11.3) | 42.8 (12.3) | 47.1 (12.2) | 49.2 (14.3) |
| **Baseline PCE score, median [IQR], %** | 9.0 [7.0, 13.0] | 7.0 [6.0, 10.0] | 15.0 [10.0, 21.0] | 8.0 [6.0, 11.0] | 8.0 [6.0, 10.0] | 10.0 [7.0, 15.0] | 12.0 [8.0, 19.5] |
| **Statin usage, n (%)** | 29 (0.6) | 55 (14.6) | 36 (1.9) | 154 (40.6) | 46 (19.7) | 63 (14.8) | 481 (17.7) |
| **Hs-CRP, median [IQR], mg/L** | 2.4 [1.1, 5.4] | 1.7 [0.8, 4.0] | 2.2 [1.1, 3.8] | 1.8 [0.8, 3.6] | 1.9 [0.8, 3.9] | 0.3 [0.1, 0.5] | 2.2 [1.0, 4.5] |
| **eGFR, mean (SD), mL/min/1.73m^2^** | 100.2 (15.3) | 102.3 (25.9) | 82.5 (17.3) | 68.8 (16.2) | 97.5 (12.8) | 93.3 (19.4) | 77.2 (15.4) |
| **Assessments time interval, mean (SD), years** | 6.0 (0.3) | 4.9 (0.4) | 4.9 (0.1) | 4.4 (0.5) | 6.1 (0.6) | 4.6 (0.6) | 4.8 (0.3) |

^a^ The percentage may not sum to 100% because of missing data.

Abbreviations: ASCVD**,** atherosclerotic cardiovascular disease; SD, standard deviation; BMI, body mass index; SBP, systolic blood pressure; HDL-C, high-density lipoprotein cholesterol; PCE, pooled cohort equation; IQR, interquartile range; hs-CRP, high-sensitive C-reactive protein; eGFR, estimated glomerular filtration rate.

Figure S2. Associations between the 5-year ASCVD risk status change patterns and subsequent ASCVD incidence stratified by age, sex, and race (with consistently high as the reference).

* *p* for interaction < 0.05

All HRs were adjusted for educational attainment and family income, body mass index, high sensitive C-reactive protein, statin usage, and estimated glomerular filtration rate.

Abbreviations: ASCVD, atherosclerotic cardiovascular disease; HR, hazard ratio; CI, confidence interval.

Figure S3. Associations between the 5-year ASCVD risk status change patterns and subsequent ASCVD incidence stratified by age, sex, and race (with consistently non-high as the reference).

* *p* for interaction < 0.05

All HRs were adjusted for educational attainment and family income, body mass index, high sensitive C-reactive protein, statin usage, and estimated glomerular filtration rate.

Abbreviations: ASCVD, atherosclerotic cardiovascular disease; HR, hazard ratio; CI, confidence interval.

Figure S4. Hazard ratios and 95% confidence intervals for incident atherosclerotic cardiovascular disease per 5% decrease in PCE risk score over 5-year intervals stratified by risk change patterns


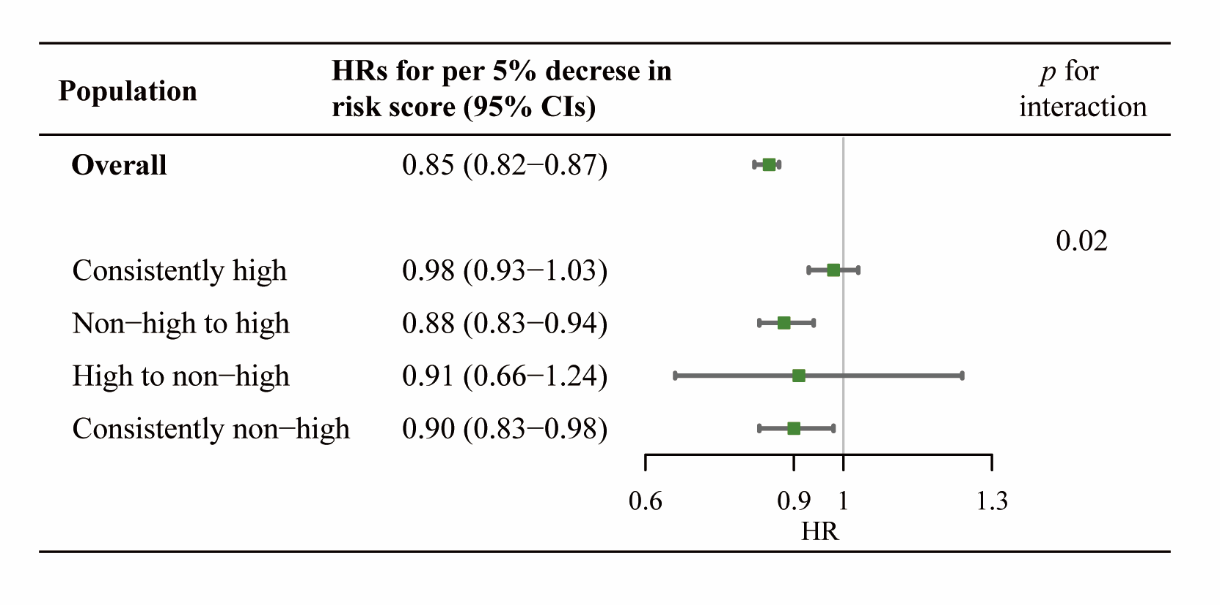


All HRs were adjusted for educational attainment and family income, body mass index, high sensitive C-reactive protein, statin usage, and estimated glomerular filtration rate.

Figure S5. Hazard ratios and 95% confidence intervals for incident atherosclerotic cardiovascular disease per 5% increase in PCE risk score over 5-year intervals stratified by risk change patterns


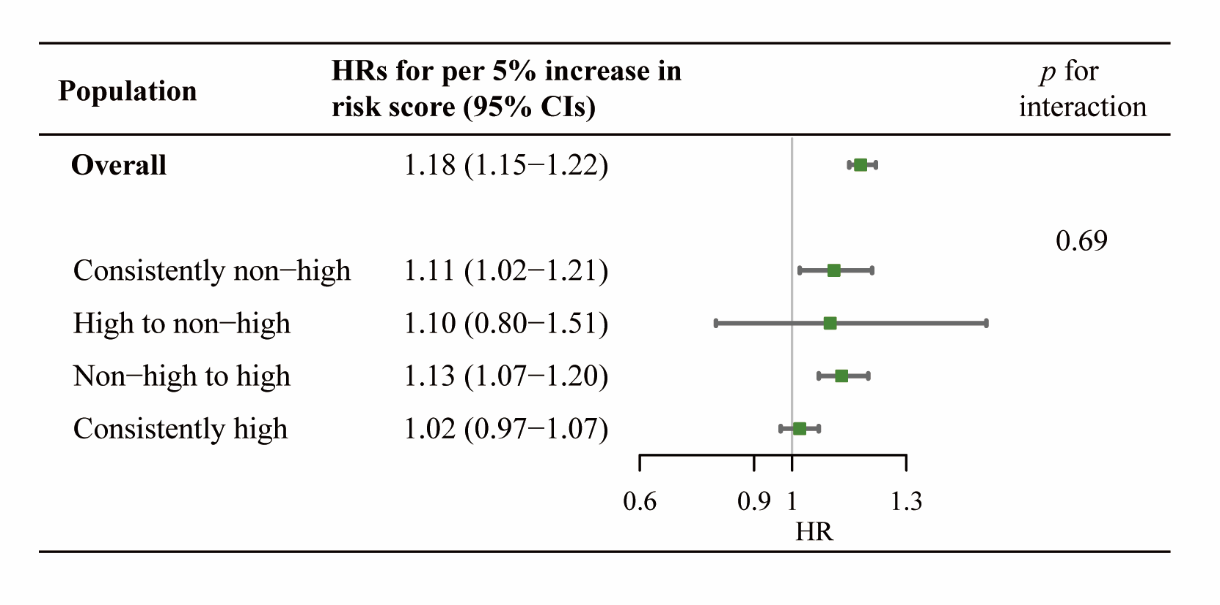


All HRs were adjusted for educational attainment and family income, body mass index, high sensitive C-reactive protein, statin usage, and estimated glomerular filtration rate.

Table S2. Sensitivity analysis by excluding participants with ASCVD incidence that occurred during the first 3-year follow-up

|  | **Events/Total** | **Multivariable model ^a^** | |
| --- | --- | --- | --- |
|  |  | **HR (95% CI)** | ***p* value** |
| **ASCVD risk change group (with consistently high as the reference)** |  |  |  |
| Consistently high | 380/1400 | 1 (Reference) | / |
| Non-high to high | 542/2091 | 0.67 (0.58-0.78) | <0.01 |
| High to non-high | 36/206 | 0.55 (0.37-0.80) | <0.01 |
| Consistently non-high | 1133/6860 | 0.34 (0.29-0.38) | <0.01 |
| **ASCVD risk change group (with consistently non-high as the reference)** |  |  |  |
| Consistently non-high | 1133/6860 | 1 (Reference) | / |
| High to non-high | 36/206 | 1.63 (1.12-2.37) | 0.01 |
| Non-high to high | 542/2091 | 1.99 (1.78-2.23) | <0.01 |
| Consistently high | 380/1400 | 2.98 (2.61-3.41) | <0.01 |

^a^ Adjusted for educational attainment and family income, body mass index, high sensitive C-reactive protein, statin usage, and estimated glomerular filtration rate.

Abbreviations: ASCVD, atherosclerotic cardiovascular disease; HR, hazard ratio; CI, confidence interval.

Table S3. Sensitivity analysis by excluding participants with borderline risk (5-7.5%) at baseline

|  | **Events/Total** | **Multivariable model ^a^** | |
| --- | --- | --- | --- |
|  |  | **HR (95% CI)** | ***p* value** |
| **ASCVD risk change group (with consistently high as the reference)** |  |  |  |
| Consistently high | 519/1539 | 1 (Reference) | / |
| Intermediate to high | 639/2097 | 0.68 (0.60-0.78) | <0.01 |
| High to intermediate | 51/221 | 0.57 (0.41-0.79) | <0.01 |
| Consistently intermediate | 832/3954 | 0.41 (0.36-0.46) | <0.01 |
| **ASCVD risk change group (with consistently** **intermediate as the reference)** |  |  |  |
| Consistently intermediate | 832/3954 | 1 (Reference) | / |
| High to intermediate | 51/221 | 1.40 (1.01-1.94) | 0.04 |
| Intermediate to high | 639/2097 | 1.67 (1.49-1.88) | <0.01 |
| Consistently high | 519/1539 | 2.45 (2.16-2.78) | <0.01 |

^a^ Adjusted for educational attainment and family income, body mass index, high sensitive C-reactive protein, statin usage, and estimated glomerular filtration rate.

Abbreviations: ASCVD, atherosclerotic cardiovascular disease; HR, hazard ratio; CI, confidence interval.
